# Supplementary material for: Molecular evidence of hybridization between pig and human Ascaris indicates an interbred species complex infecting humans
Source: eLife. 2020 Nov 6;9:e61562. doi: 10.7554/eLife.61562 (PMC7647404; doi:10.7554/eLife.61562)
Supplement: Supplementary file 6. — Whether all sequences collected globally, or just sequences collected in Kenya as part of this study were examine, the Tajima’s D value was negative and significant (indicating an excess of low frequency polymorphisms) and the Fu’s Fs was positive but not significant (potentially indicating a deficiency in diversity as would be expected in populations that has recently undergone a bottle neck event). [file elife-61562-supp6.docx]

| Population | No. Sep | No. haps | Tajima’s D |  | Fu’s F |  |
| --- | --- | --- | --- | --- | --- | --- |
| Global | 85 | 48 | -1.5691 | (P 0.028) | 8.5673 | (P 0.975) |
| Kenya | 68 | 35 | -1.28930 | (P 0.079) | 4.979 | (P 0.917) |

Table S7. Demographic analyses using Tajima’s D and Fu’s F statistic across complete mitochondrial genomes as a detection for the signature of population expansion events
